# Effectiveness of a 6-Month 22.5-mg Leuprolide Acetate Depot Formulation With Tamoxifen for Postoperative Premenopausal Estrogen Suppression in Hormone Receptor-Positive Breast Cancer

**DOI:** 10.3389/fonc.2021.665426

**Published:** 2021-04-28

**Authors:** Zhen-Yu Wu, Young-jin Lee, Heejeong Kim, Jongwon Lee, Il Yong Chung, Jisun Kim, Saebyeol Lee, Byung-Ho Son, Sung-Bae Kim, Jae Ho Jeong, Gyungyub Gong, Sei-Hyun Ahn, BeomSeok Ko

**Affiliations:** ^1^ Department of Breast Surgery, Shanghai East Hospital, Tongji University School of Medicine, Shanghai, China; ^2^ Division of Breast Surgery, Department of Surgery, Asan Medical Center, University of Ulsan College of Medicine, Seoul, South Korea; ^3^ Department of Oncology, Asan Medical Center, University of Ulsan College of Medicine, Seoul, South Korea; ^4^ Department of Pathology, Asan Medical Center, University of Ulsan College of Medicine, Seoul, South Korea

**Keywords:** breast neoplasms, premenopausal, endocrine therapy, leuprolide acetate, hormonal receptor positive

## Abstract

**Background:**

In patients with hormone receptor-positive (HR+)/premenopausal breast cancer, luteinizing hormone-releasing hormone analogs (LHRHas) are used as standard endocrine treatment. Based on previous clinical studies, 1-month formulations are recommended in most breast cancer treatment guidelines, but long-acting formulations facilitate reductions in side effects and patient discomfort caused by frequent administration. However, few efficacy studies have been conducted on 6-month formulations. Therefore, this study aimed to evaluate the efficacy of 6-month formulations of LHRHas.

**Methods:**

This retrospective study was conducted from January 2018 to December 2019 and involved premenopausal patients with HR+ breast cancer administered 6-month LHRHas as adjuvant treatment after surgery, and those previously administered chemotherapy or other LHRHa types were excluded. Patients’ estradiol (E2) and follicle-stimulating hormone (FSH) levels were measured before surgery, and their E2 levels were also measured at 3, 6, 12, 18, and 24 months at periodic postsurgical examinations.

**Results:**

A total of 228 patients were included, and the median patient age was 44 (range, 25–54) years. The mean serum E2 and FSH levels before surgery were 69.7 (range, 4–683) pg/mL and 7.3 (range, 0.4–88.9) mIU/mL, respectively, whereas the mean serum E2 level monitored at intervals during the 6-month LHRHa administration was 5.5 (range, 4.0–52) pg/mL. No women menstruated during the follow-up period after the LHRHas administration, and the E2 levels were less than 30 pg/mL in all patients except one.

**Conclusions:**

The 6-month LHRHa formulation adequately suppressed ovarian function in premenopausal patients with HR+ breast cancer. This indicates that long-acting LHRHas can be effectively used for patient convenience and that there is high compliance with long-term use.

## Introduction

Breast cancer is the most prevalent malignancy among women worldwide, and approximately 75% of patients are hormone receptor-positive (HR+) (1). HR+ breast cancer is driven by female hormones, which promote cancer cell growth; thus, endocrine treatment is administered as the standard treatment (2). Several types of surgical and medical treatments have been used to reduce female hormone levels to suppress the progression of cancer and prevent recurrence. In premenopausal women, reducing the production of estradiol (E2) by suppressing ovarian function is the basis of treatments such as bilateral oophorectomy, ovarian irradiation, and the administration of the synthetic analog of luteinizing hormone (LH)-releasing hormone analogs (LHRHas) (3).

At the beginning of LHRHa administration, anterior pituitary LHRH receptors are stimulated to increase the production of LH and follicle-stimulating hormone (FSH), which increases E2 levels. However, long-term LHRHa administration desensitizes and downregulates these receptors, which inhibits LH, FSH, and E2 production (4). LHRHas show ovarian suppressive effects equivalent to those of oophorectomy; thus, according to several recently updated breast cancer adjuvant treatment guidelines, the administration of ovarian function suppression (OFS) therapy after chemotherapy is recommended in high-risk premenopausal patients with HR+ breast cancer (2, 4–6).

Several past clinical trials have documented that LHRHas can be used as an alternative postoperative adjuvant therapy to chemotherapy in premenopausal women with HR+, human epidermal growth factor receptor 2 (HER2)-negative, and lymph node +/- breast cancer (7–13).

Attempts are being made to use LHRHas instead of chemotherapy in patients who are expected to respond poorly to chemotherapy. In most previous OFS-related clinical studies, an LHRHa was administered as a 1-month regimen (3.75 mg of leuprolide, 3.6 mg of goserelin, or 3.75 mg of triptorelin), and most breast cancer treatment guidelines also recommend 1-month treatment intervals (2, 5, 6, 14, 15).

However, to reduce the associated side effects, discomfort, and inconvenience, drug dosage regimens at 3-month or 6-month (6M) intervals have been developed for treatment, and several clinical studies have demonstrated no difference in their safety and effectiveness compared with 1-month regimens (11, 16–19). The anticancer activity of LHRHas is inherently aimed at effectively suppressing E2 levels to postmenopausal levels. The serum E2 concentrations of postmenopausal women are generally < 30 pg/mL (11, 20), and previous studies have shown that LHRHas administrated at 1- and 3-month intervals effectively suppress the E2 concentration of premenopausal patients with breast cancer to postmenopausal levels (16, 17).

The purpose of this study was to retrospectively observe patients’ E2 levels during cotreatment with an injectable LHRHa 6M formulation (22.5 mg of leuprolide) plus tamoxifen to determine the safety and effectiveness of the treatment.

## Materials and Methods

This was a retrospective study involving patient chart review, which was approved by the Institutional Review Board (IRB) of Asan Medical Center (IRB No. 2017-1341). We enrolled LHRHa-treated premenopausal women with HR+ breast cancer who had undergone related surgery at Asan Medical Center between January 2018 and December 2019. Among the patients administered the various LHRHa regimens, only those who received a 6M formulation of 22.5 mg of leuprolide (Leuplin^®^ 22.5 mg, Takeda Pharmaceutical Company) with tamoxifen (oral, 20 mg/day) were included in the analysis. Patients who underwent a previous bilateral oophorectomy were excluded because the ovarian function of these patients may have already been suppressed. Patients who received chemotherapy or had metastatic disease were also excluded because the systemic treatment for distant metastasis may be a confounding factor in the analysis of E2 levels. Leuplin^®^ 22.5 mg was recognized by the Ministry of Food and Drug Safety and administered to patients under the National Health Insurance Policy of the Republic of Korea. Basic patient characteristics such as age and body mass index (BMI) were recorded, and the type of cancer, lymph node metastasis, and stage were determined based on the pathological results. The patients underwent mastectomy or conserving surgery according to the size of the tumor and received adjuvant therapy in accordance with breast cancer guidelines at the treating physician’s discretion.

The administration of LHRHas was determined by the treating physician and was planned for 2–5 years, depending on the disease stage and age of the patient; patients were defined as premenopausal if menstruation had occurred within 1 year or if the E2 level was > 30 pg/mL and the FSH level was < 30 mIU/mL at the preoperative examination (21).

The effectiveness of the treatment was measured by cessation of menstruation and the E2 level. The FSH level was used to determine whether menopause had occurred before surgery; however, the administration of tamoxifen alters FSH levels (21, 22); therefore, the FSH level cannot be used as an index of the effect of LHRHas. The menstrual status and E2 levels (limit of quantification of 4.0 pg/mL) were monitored at 3, 6, 12, 18, and 24 months after surgery during each patient’s visit cycle. The patients’ clinical information and pathological test results were analyzed. In addition, the preoperative E2 levels and episodes of drug discontinuation due to side effects during the administration of the 6M formulation were retrospectively investigated. Normal menstruation was determined based on a clinical examination of the patients in the obstetrics and gynecology department. Cases of vaginal bleeding in which the volume was considerably less than that of normal menstruation were not considered menstruation.

## Results

**Figure 1:**
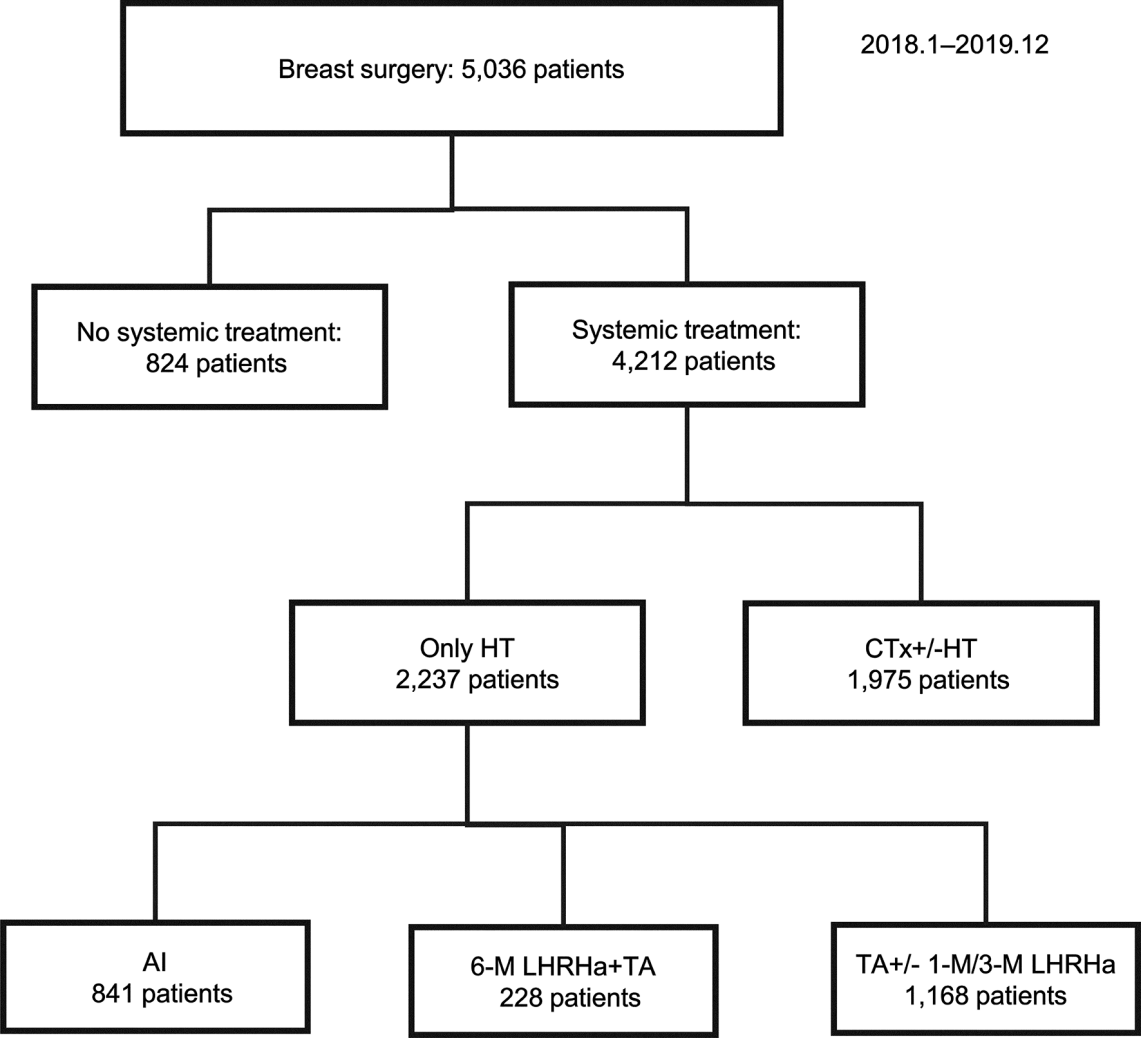
Summary of enrolled patients. AI, aromatase inhibitor; CTx, chemotherapy; HT, hormonal therapy; LHRHa, luteinizing hormone-releasing hormone analog; M, month; TA, tamoxifen.

From January 2018 to December 2019, 5,036 patients underwent surgery for breast cancer, including 350 who received a 6M LHRHa as adjuvant therapy. Among these patients, we excluded 122 who received chemotherapy or other OFS treatments; thus, a total of 228 patients were included in the analysis (**Figure 1**). The patient and tumor characteristics are shown in **Table 1**. The median patient age was 44 (range, 25–54) years. The mean BMI was ≤ 24 kg/m (2) in 172 (75.4%) patients. The cancer type was invasive ductal, invasive lobular, and other classified cancers in 189 (82.9%), 19 (8.3%), and 20 (8.8%) patients, respectively. Furthermore, 137 (60.1%), 89 (39.0%), and 2 (0.9%) patients had stage I, II, and III cancer, respectively.

**Table 1:** Patient and tumor characteristics.

Patients (N = 228)	N (%)
Age (years)	
< 35	25 (11.0)
35–39	29 (12.7)
40–44	75 (32.9)
45–50	90 (39.5)
> 50	9 (3.9)
Body mass index (kg/m^2^)	
≤ 24	172 (75.4)
> 24	56 (24.6)
Pathology	
Invasive ductal carcinoma	189 (82.9)
Invasive lobular carcinoma	19 (8.3)
Other	20 (8.8)
Stage	
I	137 (60.1)
II	89 (39.0)
III	2 (0.9)
T stage	
T1	161 (70.6)
T2	62 (27.2)
T3	5 (2.2)
N stage	
N0	183 (80.3)
N1	43 (18.9)
N2	2 (0.8)
N3	0 (0)
Tumor grade	
I	16 (7.0)
II	198 (86.8)
III	14 (6.2)
Multifocal tumors	
Yes	158 (69.3)
No	70 (30.7)
Subtype	
HR+, HER2-	225 (98.7)
HR+, HER2+	3 (0.3)
HR-, HER2+	0 (0)
HR-, HER2-	0 (0)

HER2, human epidermal growth factor receptor 2; HR, hormone receptor.

**Figure 2:**
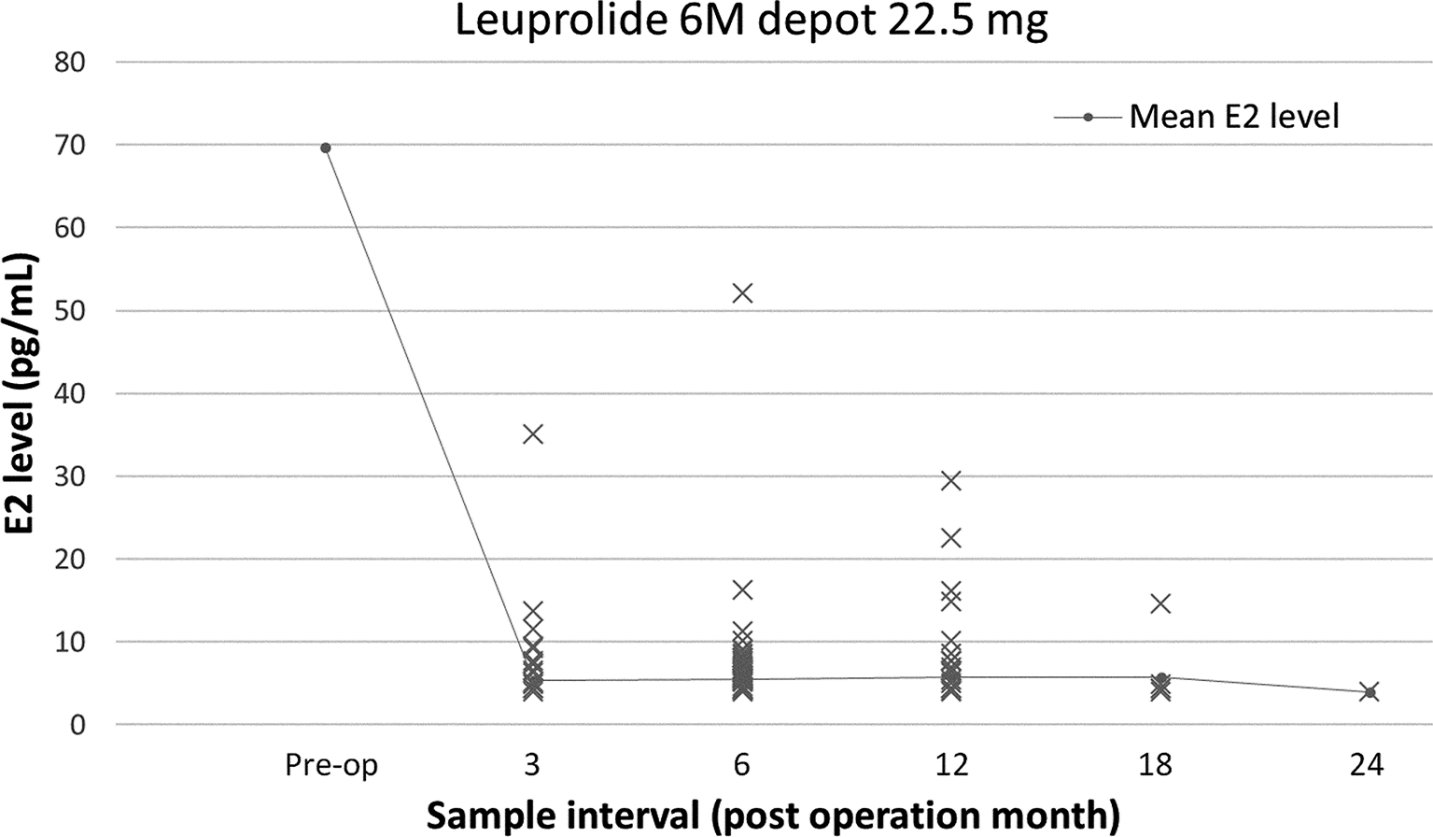
Changes in serum estradiol (E2) levels before and after administration of a luteinizing hormone-releasing hormone analog (LHRHa, total).

Breast-conserving surgery and mastectomy were performed in 177 (77.6%) and 51 (22.4%) patients, respectively, and 215 (94.3%) and 13 (5.7%) patients underwent sentinel node biopsy and axillary lymph node dissection, respectively (**Table 2**). The preoperative examination showed mean serum E2 and FSH levels of 69.7 (range, 4.0–683) pg/mL and 7.3 (range, 0.4–88.9) mIU/mL, respectively. The mean serum E2 levels over the entire dosing period was 5.5 (range, 4.0–52) pg/mL. The changes in serum E2 levels before and after administration of the 6M LHRHa are shown in **Table 3** and **Figure 2**. The suppression rate of the serum E2 level to the menopausal level (< 30 pg/mL) during treatment was 99.6%. The mean E2 level in young patients (age ≤ 40 years) and older patients (age > 40 years) was 6.4 ± 7.5 pg/mL and 5.2 ± 3.1 pg/mL, respectively (*P* = 0.09). During the follow-up period, 12 patients (5.3%) experienced vaginal bleeding, which was not considered normal menstruation because the volume was considerably less than that of normal menstruation and because their serum E2 levels were also considered not be related to menstruation. Accordingly, no patient menstruated during the follow-up period after OFS therapy in this study. The serum E2 levels were < 30 pg/mL in all patients except for one. The only patient who showed an E2 level > 30 pg/mL after OFS therapy was 35 years old with a BMI that ranged between 22.7 and 23.4 kg/m (2) during the follow-up period. The preoperative E2 concentration of this patient was 140 pg/mL, and her E2 concentrations 3, 6, and 12 months after surgery were 35, 52, and 16.1 pg/mL, respectively. No serious side effects were observed that necessitated discontinuation of OFS therapy or changes in treatment during the observation period.

**Table 2:** Type of surgery and adjuvant treatment.

Total patients (N = 228)	N (%)
Surgery	
Mastectomy	51 (22.4)
Breast-conserving surgery	177 (77.6)
Axillary surgery	
Sentinel lymph node biopsy	215 (94.3)
Axillary lymph node dissection	13 (5.7)
Radiation therapy	
Yes	181 (79.4)
No	47 (20.6)

**Table 3:** Changes in serum estradiol levels before and after administration of a 6-month luteinizing hormone-releasing hormone analog.

	Pre-op (n = 226)	3 months(n = 60)	6 months(n = 109)	12 months(n = 56)	18 months(n = 7)	24 months(n = 1)
Mean (SD), pg/mL	69.7 (91.9)	5.3 (4.4)	5.5 (4.9)	5.8 (4.7)	5.7 (3.9)	4.0 (-)
Median (range), pg/mL	34.8 (4.0–683)	4.0 (4.0–35)	4.0 (4.0–52)	4.0 (4.0–29.4)	4.3 (4.0–14.6)	NA

SD, standard deviation; NA, not applicable.

The distribution of E2 levels widened at 12 months during the OFS therapy compared with the other points in time in this study (**Figure 2**). We tried to analyze patient factors (age ≤40 vs. >40 years; BMI ≤24 vs. >24 kg/m (2); preoperative E2 level ≤80 vs. >80 pg/mL) associated with an E2 level >4.0 pg/mL at 12 months after the LHRHas administration and found that a preoperative E2 level >80 pg/mL was the only factor significantly associated with an E2 level >4.0 pg/mL at 12 months (23.7% vs. 53.6%; *P*=0.019).

## Discussion

Breast cancer has a variety of subtypes with different treatments and prognoses. Optimized treatment based on tumor biology is important, and endocrine treatment plays an important role in patients with HR+ tumors (2, 23). Tamoxifen is a selective estrogen receptor modulator that is used as a basic endocrine treatment in pre- and postmenopausal women. Surgical oophorectomy was first reported in 1889, and the level of circulating estrogens was clearly reduced to menopausal levels. However, the morbidity and mortality associated with general anesthesia and surgery have become a problem (24). LHRHas are the most commonly used OFS treatments because they are associated with fewer complications than oophorectomy or ovarian irradiation and have the advantage of temporarily inhibiting ovarian function (3). LHRHas also have fewer side effects, such as hair loss, infection, anemia, and vomiting, than chemotherapy, and patients recover from any side effects quickly when administration is stopped, making this treatment more convenient for patients with poor systemic conditions and thus lowering the rate of discontinuation (7–9).

Based on the advantages of LHRHas, several studies have replaced chemotherapy as breast cancer adjuvant therapy (7, 8). The Austrian Breast and Colorectal Cancer Study Group (ABCSG) trial 5 compared 3 years of goserelin plus 5 years of tamoxifen and 6 cycles of cyclophosphamide, methotrexate, and fluorouracil (CMF) in 1,034 patients with stage I and II breast cancer, and the endocrine therapy group showed a longer relapse-free survival period than the CMF group (7). The results of the Zoladex Early Breast Research Association (ZEBRA) trial of goserelin and CMF with a median follow-up period of up to 6 years in premenopausal patients who were node-positive showed no difference in the disease-free survival (DFS) in both groups, enabling LHRHas to replace the chemotherapy (8).

In breast cancer, mainstream chemotherapy has been changed from CMF to adriamycin/cyclophosphamide (AC), and the difference between OFS therapy and AC has been studied (12, 13, 25). Kim et al. (12) compared the prognosis between 318 and 269 patients in a OFS+tamoxifen and chemotherapy+tamoxifen groups, respectively, over a median follow-up period of 30 months and found no difference in the DFS (12). Sohn et al. (13) analyzed two groups of patients, an OFS+tamoxifen (n = 260) and a sequential AC+tamoxifen (n = 260) group, among 994 patients with T1-2N0, HR+ HER2- breast cancer with propensity score matching and inverse probability weighting. A comparison of the prognosis over a median follow-up period of 7.4 years showed no difference between the two groups (13). Sa-Nguanraksa et al. (25) administered 10.8 mg of goserelin (for 2 to 3 years at 3-month intervals) and tamoxifen to one group (n = 40) and AC+tamoxifen to another group (n = 130) of patients with HR+ breast cancer with a tumor size ≤ 3 cm. The study results showed that, after a 77-month median follow-up period, the DFS was not different between the two groups, and the endocrine group showed a better quality of life than the other group (25). To date, most clinical studies of OFS therapy have been conducted with a 1-month administration regimen (7, 9, 14, 15). In accordance with the results of these clinical studies, relevant guidelines recommend OFS therapy every month (2, 5, 6). However, frequent injections increase the patient’s risk of pain, hematoma formation, and infection, and frequent clinic visits are also inconvenient to the patient and thus lead to decreased treatment compliance (26). To reduce the discomfort caused by frequent injections, long-acting LHRHas were developed, and data analyses from several clinical trials have demonstrated its safety and effectiveness (11, 16, 17, 27).

Schmid et al. (11) compared the prognosis of patients treated with a 3-month depot LHRHa (n = 299) with that of patients treated with CMF (n = 300) in a randomized clinical trial with pre- or perimenopausal women with estrogen receptor-positive and lymph node-positive breast cancer. The results showed no difference in recurrence-free survival between the two groups with a median follow-up time of 5.8 years, and there were fewer side effects, such as nausea, vomiting, and alopecia, in the OFS group than in the other group (11). Masuda et al. (16) reported the area under the concentration-time curve (AUC) of E2 following treatment with a 3-month 10.8-mg depot of goserelin (n = 86) and a monthly 3.6-mg dose (n = 84) in premenopausal patients with estrogen receptor-positive breast cancer. The mean AUCs of E2 were not different between the two groups, and the safety and tolerability were not different (16). Noguchi et al. (17) conducted a phase 3, open-label, multicenter trial involving premenopausal women with estrogen receptor-positive breast cancer who were administered a 3-month 10.8-mg dose (n = 109) and a monthly 3.6-mg dose of goserelin (n = 113). The mean serum E2 concentrations at week 24 were similar between the two groups, and there was no difference in the progression-free survival (17). Kendzierski et al. (19) reported studied OFS therapy plus an aromatase inhibitor, and their retrospective review comparing 1-month (n = 100) versus 3-month dosages of (n = 101) an LHRHa + aromatase inhibitor showed no difference in the E2 levels after 3 months of administration (19). Most patients visit our clinic every 6 months according to the guidelines for breast cancer treatment; thus, prior consideration was given to 6 months of LHRHa administration in patients undergoing OFS therapy. However, currently, there are few studies on 6M treatment regimens with LHRHas in patients with breast cancer. Kurebayashi et al. (18) compared 6M and 3-month LHRHa treatments in a phase III, randomized, open-label, parallel-group comparative study. The E2 suppression rate was 97.6% and 96.4% in a total of 167 patients treated for 6 (n=83) and 3 (n=84) months, respectively, and there was no difference in the safety profiles or tolerability (18). In contrast to chemotherapy, LHRHas have no direct cytotoxicity, and their primary function is OFS.

The results of several studies indicate that long-acting LHRHas effectively suppress ovarian function, based on measurements of the E2 levels, similar to short-acting agents (16–19). Our study of 6M LHRHa+tamoxifen-treated patients showed that the E2 levels measured after drug administration were mostly in the menopausal range. The causes of and risk factors for OFS failure are not well understood. In the group of patients who received exemestane plus triptorelin in the Suppression of Ovarian Function Trial Estrogen Substudy (SOFT-EST), E2 levels above the baseline were associated with a high BMI (*P* = 0.05) (28). de Ciantis et al. (29) reported that a young age (< 40 years) may cause suppression failure. In the present study, the mean E2 level in patients aged ≤40 years was higher than that in patients aged >40 years, with borderline statistical significance (6.4 ± 7.5 pg/mL vs. 5.2 ± 3.1 pg/mL; *P* = 0.09) during the treatment periods. This result reflects that the suppressive effect of LHRHas on E2 levels may differ in premenopausal patients of different age groups. However, when we analyzed the patient factors associated with an E2 level >4.0 pg/mL at 12 months, a preoperative E2 level >80 pg/mL was the only factor significantly associated with an E2 level >4.0 pg/mL at 12 months, and traditional factors, including patient age and BMI, were not associated with an E2 level >4.0 pg/mL at 12 months, probably due to the small number of cases in this subgroup. In our study, the E2 level exceeded 30 pg/mL in only one patient during the treatment period. This patient was 35 years old at the time of diagnosis and had a BMI between 22.7 and 23.4 kg/m^2^ during the follow-up period. In several studies on OFS, ovarian escape cases were observed during OFS administration (28, 30); however, to our knowledge, to date, no considerable results have shown the prognostic significance of ovarian escape, and currently, there is no guideline for such cases. Accordingly, careful follow-up and further investigations are needed for ovarian escape cases.

There are some limitations to the present study. First, this was a retrospective, single-center cohort study with no control or comparator. Second, the E2 level was not measured in all patients during treatment. Third, systematic analyses of adverse effects, patient satisfaction during treatment, and survival outcomes were not conducted. Despite these limitations, our study showed that 6M LHRHas effectively inhibited ovarian function.

## Conclusion

Our study demonstrated that, in premenopausal patients with HR+ breast cancer, administering a 6M LHRHa formulation after surgery effectively suppressed ovarian function. Consequently, long-acting LHRHas are a good adjuvant treatment option based on the decreased need for numerous injections leading to patient inconvenience and discomfort.

## Data Availability Statement

The original contributions presented in the study are included in the article/supplementary material. Further inquiries can be directed to the corresponding author.

## Ethics Statement

The studies involving human participants were reviewed and approved by the institutional review board of Asan Medical Center, Seoul, Korea (No. 2017-1341). The ethics committee waived the requirement of written informed consent for participation.

## Author Contributions

Z-YW and Yj-L contributed equally to this work and should be considered co-first authors. All authors contributed to the study conception and design. Material preparation, data collection, and data analysis were performed by Z-YW and Yj-L. The first draft of the manuscript was written by Z-YW and Yj-L, and all authors commented on previous versions of the manuscript. All authors contributed to the article and approved the submitted version.

## Conflict of Interest

The authors declare that the research was conducted in the absence of any commercial or financial relationships that could be construed as a potential conflict of interest.
